# How Does the Effort Spent to Hold a Door Affect Verbal Thanks and Reciprocal Help?

**DOI:** 10.3389/fpsyg.2015.01737

**Published:** 2015-11-12

**Authors:** Glenn R. Fox, Helder Filipe Araujo, Michael J. Metke, Chris Shafer, Antonio Damasio

**Affiliations:** Department of Psychology, Brain and Creativity Institute, Dornsife College of Letters, Arts and Sciences, University of Southern CaliforniaLos Angeles, CA, USA

**Keywords:** social influence, social cognition, social behavior, cooperation, interpersonal interaction

## Abstract

When someone holds a door for us we often respond with a verbal “thanks.” But given such a trivial favor, our feelings can vary considerably depending on how the door is held. Studies have shown that verbal thanking increases in relation to door-holding effort. However, it is unclear how such a favor can lead to verbal thanks in addition to reciprocal help. We examined how holding a door in an effortful or non-effortful manner relates to verbal thanking and reciprocal helping. We measured: (1) whether participants verbally thanked the experimenter, (2) whether they agreed to help another person by taking a survey, and (3) whether they helped pick up objects (pens) that the door-holder subsequently dropped. Participants in the effortful condition were more likely to offer verbal thanks, to help pick up the pens, and to walk a greater distance to pick them up. Participants who thanked the door-holder, however, were not more likely to provide help.

## Introduction

When someone holds a door for us, we generally say “thank you” and go about our day. This seemingly trivial encounter, however, can inspire a wide range of reactions contingent on the manner in which the door is held. Imagine facing a door-holder who is smiling, effortfully opening the door and letting you walk through before she enters (high-effort). Or imagine that she lazily holds the door and non-effortfully props it with an outstretched arm while staring at a text message (low-effort). We save energy in both cases, but the emotional feeling and the reaction generated by each case can be quite different. Measurement of such reactions can reveal finely tuned mechanisms for understanding others, and for understanding how we choose to respond in kind. We can examine whether door-holding is an extension of our ability to feel gratitude for favors based on the genuine helpfulness of the favor (Wood et al., 2008). This study sought to examine how effort spent holding a door (a) leads to verbal thanks, (b) changes the likelihood of reciprocal helping, and (d) whether verbal thanks predicted subsequent reciprocal helping behavior.

In the realm of favors, the act of door-holding must rank near the smallest we can receive, yet it reveals important social customs and psychological mechanisms (Santamaria and Rosenbaum, 2011). We define favor broadly, as a kind or helpful act for another person. For instance, verbal thanks may be regarded as a rote social norm to be performed for every favor, but there is evidence that people are more likely to verbally thank a benefactor holding a door in an effortful, polite manner, than if the door was held in a casual manner (Goldman et al., 1981; Okamoto and Robinson, 1997). Verbal thanks for door-holding may indeed complete the duty for recognizing the door-holder's effort, indicating full recognition of the favor and an end to the interaction. It is also possible that verbal thanks indicate a feeling of gratitude that can manifest a desire to repay the favor through an effortful gesture (McCullough et al., 2001). Regardless of whether verbal thanking *should* be associated with actual helping behavior, the relationship between verbal thanking and helping has not been studied.

We frame this study in the broader context of research on gratitude. In general, it is known that receiving favors, and the gratitude they produce, can lead to reciprocal helping directed to the benefactor or to others (Bartlett and DeSteno, 2006; Nowak and Roch, 2007). Repayment for favors pivots in part around how we perceive the donor's own effort to provide the favor (Tsang, 2006a). The amount of reciprocated help is proportional to the amount of prior help received (Wilke and Lanzetta, 1970), and to the amount of perceived effort put into providing a favor (Regan, 1971; Greenberg and Frisch, 1972; Goei and Boster, 2005; Tsang, 2007). However, the principles learned from these studies have rarely been applied to field observations of natural behavior. Likewise, previous observational studies have not examined if door-holding can inspire reciprocal help (Goldman et al., 1981; Okamoto and Robinson, 1997; Santamaria and Rosenbaum, 2011; McCarty and Kelly, 2013). Reciprocal helping, by our definition, refers both to help directed toward a stranger, i.e., upstream reciprocity (Bartlett and DeSteno, 2006; Nowak and Roch, 2007; McCullough et al., 2008), and to help directed to the door-holder himself, i.e., the norm of reciprocity (Gouldner, 1960).

The genuine helpfulness of a gift is an important factor for gratitude (Algoe et al., 2008; Wood et al., 2008). For this investigation, we consider effort to be an umbrella term encompassing the factors related to the act of holding the door. In this study, we are less focused on which individual factors are involved in the door-holding and more on how overall effort elicits behavior. Verbal reactions to door-holding elicit verbal recognition in proportion to the effort spent to hold the door (Okamoto and Robinson, 1997). Thus, we set out to test both the highest- and the lowest-effort forms of door-holding. Given an act of door-holding that was either passive and low-effort, or genuine and high-effort, we tested whether the recipient offered verbal thanks, and whether the recipient reciprocated the favor toward another person (i.e., agreeing to take a survey, Study I), or to the benefactor (i.e., helping the door-holder pick up accidentally spilled pens, Study II). In other words, we conceptualized effort as a combination of factors related to the general positive social effort involved in smiling and making eye contact (or not), and the extent of the behavioral effort of going out of the way to physically prop the door open. In both studies, participants' behavior was coded in terms of whether they offered verbal thanks and whether they helped with the subsequent favor request.

There are intrinsic limitations to the method of observational studies of behavior, but studies of this type can provide insight into social cognition and behavior that is not available through laboratory testing and self-report measures (Patterson, 2008). A single investigation of field behavior cannot include all the manipulations of the available factors. For instance, our investigation involves factors of effort, location, gender, ingress vs. egress from a building, and the type of favor requested of the participant. A series of studies that manipulates each of these in turn would require a very large number of experiments. As such, interpreting the findings from these experiments can be clouded by alternative explanations. However, the importance of observational studies has been stressed as a way to validate laboratory paradigms using real-world settings (Lewandowski and Strohmetz, 2009). More broadly, careful observation of trivial and mundane acts can reveal interesting and generalizable facts about emotion and social relationships (Patterson, 2008; Patterson et al., 2014), and can perhaps benefit public policy (Coxon et al., 2010).

We predicted that offering verbal thanks and reciprocal helping after having the door held would be related to distinct degrees of perceived effort put into producing the favor. We predicted that verbal thanks would be more frequent in the high-effort condition; that helping would be more frequent when the door was held in the high-effort condition compared to the low-effort condition and that participants in the high-effort condition would spend more effort to reciprocate. We also predicted that reciprocal help would be more frequent when directed toward the benefactor than toward a stranger, based on an earlier study of gratitude and reciprocal helping (Bartlett and DeSteno, 2006).

## Study I

We first investigated whether holding a door with high- or low-effort would lead a participant to offer verbal thanks and/or reciprocate by subsequently participating in a lengthy survey of personality questionnaires. We predicted that individuals who walked through a door that was held in the high-effort condition would be more likely (a) to offer verbal thanks, (b) to participate in the survey, and (c) to spend more time on the survey than those individuals for whom the door was held in the low-effort condition.

### Method

### Participants

The experimenters interacted with a total of 144 visitors to USC's Leavey Library, the participants in this study. Twenty-four trials had to be removed due to violations to the study procedure, such as the participant recognizing one of the researchers, or the participant picking up a phone immediately upon leaving the building. The final sample consisted of 120 participants, gender balanced (51 female); this sample size was chosen based on similar previous studies of reciprocal helping (Goldman et al., 1981; Okamoto and Robinson, 1997; Santamaria and Rosenbaum, 2011). Our data collection stopped when the sample size was comparable to previous studies of gratitude and reciprocal helping directed toward a stranger (Bartlett and DeSteno, 2006). There were 40 participants in each of the three conditions (high-effort, low-effort, and a control condition described below). All research activities were performed in accordance with USC's institutional review board policies concerning human subjects research. Participants were given an information sheet prior to taking the surveys and were told that the surveys were part of an experiment; they were debriefed when they finished the surveys.

### Procedure

This study involved three experimenters: (1) a door-holder, (2) an interceptor who asks the participant to take a survey, and (3) a field coordinator. It included three conditions, (1) a low-effort condition, (2) a high-effort condition, and (3) a control condition. In the low-effort condition, the door-holder waited inside the building and walked in front of the participant to hold the door for the participant as both the door-holder and the participant exited the building. The door-holder was instructed not to look back at the participant, as this had been shown to possibly induce a small verbal expression of thanks in a previous study of door-holding (Okamoto and Robinson, 1997). To prevent the participant from feeling like the door-holder cut in front, the door-holder practiced the trials and timed the door-holding such that the participant's rate and path through the door were unimpeded. In the high-effort condition, the door-holder waited outside the building, watching through the glass door into the interior of the building. When the participant was approximately 10 feet from the door, the door-holder approached the door, opened it and held it so that the participant could exit before the door-holder could enter the building. The door-holder held the door while making eye contact and smiling. In this condition, the door-holder is putting the participant's need before her own to save physical energy to the participant and provide psychological benefit through a thoughtful gesture (Algoe et al., 2013). The smiling was aimed to increase the participant's perception of genuine helpfulness, which has been shown to increase gratitude (Wood et al., 2008). The goal in using these two conditions was to maximize the separation between the emotions induced by the door-holding. We mimicked Okamoto and Robinson's (1997) conditions with the lowest and highest forms of reciprocal helping create the maximum difference in the feelings and behaviors elicited by the conditions. We did not set out to find which factor in particular, (i.e., eye contact vs. smiling), was most effective in producing reciprocal helping; effort refers to the sum of the work done to hold the door. A control condition was also used in which the door-holder did not hold the door for the participant, but the participant was still approached by the interceptor. The field coordinator handled all duties related to timing of the trials, instructing the door-holder as to the randomly selected trial condition, making sure that each experimenter was in the proper place before each trial and maintaining the on-site experimental records.

The door-holder in this experiment was a blond college-aged female. The participant in every case was a person walking on his or her own with no bystanders within a 20-foot radius. Participants were only approached if they were alone, not using a cell phone, not listening to headphones, and not carrying any items in their arms. Trials were run with a 5-min inter-trial interval. The door-holder waited for 5 min after the previous trial had concluded, then selected the second person to then leave the building. This procedure helped ensure against a selection bias in how participants were chosen.

After the participant exited the building he or she was approached by another experimenter, in this case a tall college-aged male–who was the interceptor. The interceptor stood 20 feet from the doorway with a clipboard and a stack of surveys. Before the trial, the interceptor stood facing away from the door–to prevent him from seeing the condition of the trial, thus keeping him blind to the condition of how much effort was spent to hold the door. The field coordinator stood close to the interceptor, facing the doorway to view the trial. When the door-holder identified a valid participant, she would hold the door in the appropriate condition. In the low-effort condition (where the experimenter was exiting the building in the same direction as the participant), the door-holder immediately hid behind a pillar after leaving the building to prevent being seen by the interceptor so as to not reveal to him the type of condition being conducted. The interceptor never saw the door-holder during a trial. After the door was held for a participant, the field coordinator would whisper to the interceptor a brief description of the participant (i.e., “blue shirt”), after which the interceptor would turn around and approach the participant. The interceptor would then ask the participant if he or she would like to take a survey. The interceptor was trained to say this phrase in the same way with the same intonation and body language in all trials. Both the interceptor and door-holder wore the same outfits during all data collection sessions.

**Figure 1:**
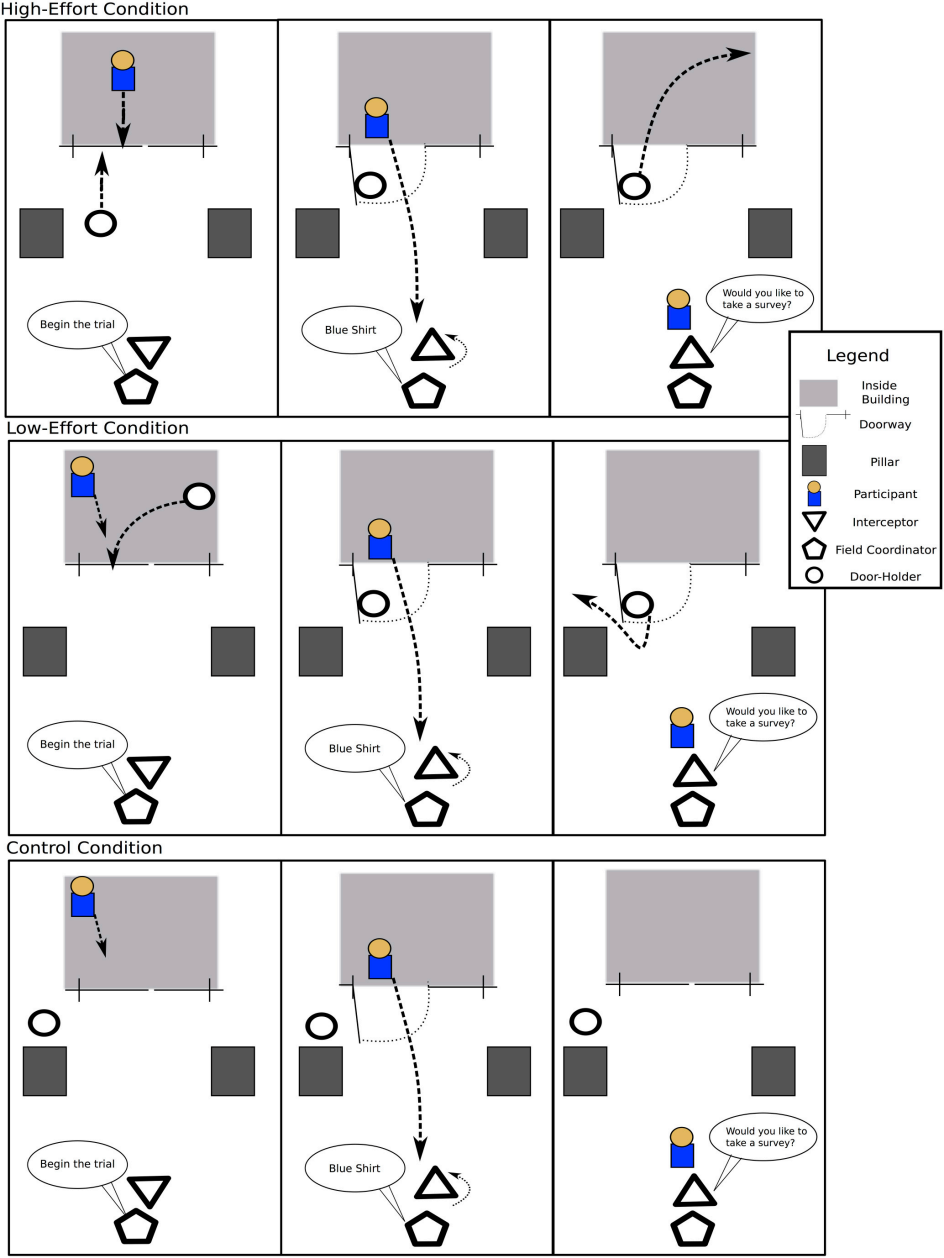
**Depiction of how trials were conducted in Study I**. The panels depict the steps for a trial in the low- and high-effort conditions, respectively, moving left to right.

The survey consisted of four common psychological questionnaires: The Eysenck Need Satisfaction scale (Lester, 1990), the six item gratitude questionnaire (GQ-6; McCullough et al., 2002), the Interpersonal Reactivity Index (IRI; Davis, 1994) and the Brief Mood Introspection Scale (BMIS; Mayer and Gaschke, 1988). We chose to include personality questionnaires as opposed to math problems, as used by Bartlett and DeSteno (2006), because we planned to examine the relationship between the personality measures and the time spent taking the survey and whether a verbal thanks was offered. Participants were told up front that they could stop the survey at any time. (See Figure 1, for a diagram detailing how these trials were conducted).

The participant's behavior was measured in terms of (a) whether or not he or she said thank you to the door-holder, (b) whether or not he or she participated in the survey, and (c) how much time he or she spent filling out the survey. The field-coordinator also recorded the sex of the participant. Statistical inference of differences in frequency of helping and verbal thanks, as well as the duration of survey taking was calculated using SPSS version 18.

### Results

Collapsing across the low-effort and high-effort conditions, 24/80 (30%) participants thanked the door holder. Participants in the high-effort condition thanked the door holder more frequently than participants in the low-effort condition χ^2^(2, 80) = 11.67; *p* < 0.001 (see Table 1, for detail).

**Table 1:** **Number of survey takers and how long they spent (in seconds) on the survey for each condition**.

**Condition**	**Thanked door-holder (out of 40)**	**Survey takers (out of 40)**	**Duration on the survey**			
			**Mean**	**Min**	**Max**	***SD***
Low-effort	5	16	496	217	770	165
High-effort	19	11	504	135	923	269
Control	N/A	12	474	26	973	275

Thirty-nine participants out of 120 agreed to take the survey. There were not any statistically significant differences in the frequency of survey-takers across conditions χ^2^(2, 120) = 1.595; *p* = 0.450 (see Table 1, for detail).

The mean duration of time spent on the survey was 491 s. The amount of time spent on the survey did not differ statistically between the low-effort and high-effort conditions *t*_(25)_ = −0.097; *p* = 0.923. Neither condition differed from control in terms of time spent on the survey [low-effort vs. control: *t*_(26)_ = 0.171; *p* = 0.795; high-effort vs. control: *t*_(21)_ = 0.264; *p* = 0.794; see Table 1, for detail].

The proportion of participants who agreed to take the surveys in the group who said thanks (41%) was not different from those in the group that did not say thanks (30%), χ^2^(2, 80) = 0.961; *p* = 0.327. Likewise, we did not observe a statistically significant relationship between offering verbal thanks and the time spent on the survey; participants who said thanks (*n* = 11; *M* = 456 s, min = 135 s, max = 923 s, *SD* = 247) did not differ statistically from those who did not say thanks (*n* = 28, *M* = 505 s, min = 26 s, max = 973 s, *SD* = 222), *t*_(37)_ = 0.601; *p* = 0.552.

### Discussion

The findings from Study I supported the prediction that verbal thanks occurs more frequently when the door is held in the high-effort condition. However, the other predictions regarding reciprocal helping were not supported. There were no differences across conditions in the frequency of reciprocal helping, measured by agreement to take the survey and time spent on the survey. Furthermore, participants who offered verbal thanks did not take the survey more frequently than those that did not offer verbal thanks.

It is possible that the absence of reciprocal helping was due to the fact that the person who held the door was not the same person asking for a favor. In Bartlett and DeSteno (2006), participants in the gratitude condition were more likely to help their original benefactor than a stranger. It may be that upstream reciprocity requires a gesture large enough to motivate us to reward others outside of the original source of goodwill (Nowak and Roch, 2007). These considerations led to the design of Study II, in which the overall effort spent to hold the door was increased, and the participants' help was directed to the door-holder.

## Study II

Similarly to Study I, an experimenter held the door for participants in a low-effort or high-effort manner. Here however, the door-holder also served as the person needing the favor from the participant. We modified the overall effort expended to hold the door; the door-holder was carrying a large filing box with a small box of pens on top. After holding the door, the experimenter fumbled the box, spilling the box of pens on the ground. This design allows a comparison to previous studies in which items were dropped and participants' helping behavior was measured (Harada and Araragi, 1981; Goldman and Fordycea, 1983; Levine et al., 1994; Monk-Turner et al., 2002; Levine, 2003; Reysen and Ganz, 2006; Vohs et al., 2006). We also manipulated the physical distance between the door-holder and the participant at the moment that the pens fell on the ground. This manipulation allowed us to measure how much effort (measured in steps necessary to return to the door-holder) participants were willing to spend to help pick up the pens in relation to the effort involved in holding the door. In other words, manipulating the distance needed to return to help with the pens allowed an additional means of parsing how the effort spent to hold the door altered the participants' behavior.

We predicted a greater frequency of verbal expressions of gratitude in response to having a door held in the high-effort condition, compared to the low-effort condition. We predicted that the frequency of reciprocal helping would decrease as the physical distance between the participant and the door-holder increased, and that the effect of distance on reciprocal helping would be more pronounced in the low-effort condition (i.e., fewer people would help in the low-effort condition at each of the decided distances). Based upon the results from Study I, we predicted that verbal thanking would not predict reciprocal helping.

### Method

### Participants

The experimenters interacted with 219 participants as they exited various buildings on USC's University Park Campus. Twenty-four participants were removed from the final dataset due to violations of the study procedure, such as talking on a cell phone or being joined by another person, leaving a total of 194 participants in the study sample. The sample size is comparable to previous studies of door-holding behavior (Goldman et al., 1981; Santamaria and Rosenbaum, 2011; McCarty and Kelly, 2013). Data collection stopped once the number of participants in the non-effortful and effortful conditions was similar to previous studies of door-holding (Okamoto and Robinson, 1997), and studies of gratitude and reciprocal helping (Bartlett and DeSteno, 2006). There were eight total conditions, (low and high-effort *x* four different distances) with 24 or 25 participants in each condition. All research activities were performed in accordance with USC's institutional review board policies concerning human subjects research.

### Procedure

**Figure 2:**
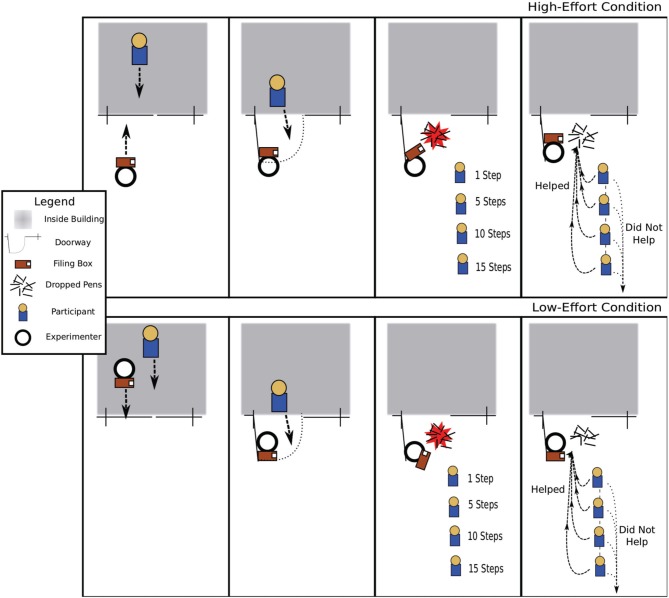
**Depiction of how trials were conducted in Study II**. The panels depict the steps for a trial in the low- and high-effort conditions, respectively, moving left to right.

In this experiment, the experimenter held the door and needed help from the participant. The door-holder in this experiment was holding a filing box with two handles on each side and a removable lid with a box containing 12 pens resting on top. To facilitate data collection, two different experimenters played the role of the door-holder/interceptor and each collected data in separate parts of campus. Both experimenters were Caucasian males, 6′3″ tall, of lean build, similar skin tone and facial bone structure, and also similar to the interceptor in Study I. The experimenter held the door for the participant in either a low-effort or high-effort condition. In the low-effort condition, the experimenter walked in front of the participant in the process of exiting a building and propped the door open with his shoulder while looking down at his cell phone. Again, the door-holder timed the door-holding such that the participant's path and speed to exit the building was unaltered. In the high-effort condition, the experimenter approached again from the outside of the building and held the door open with his free hand while the participant exited the building. (See Figure 2, for a diagram detailing how these trials were conducted).

After the door was held for the participant, the experimenter then waited as the participant walked away for a designated number of steps–1, 5, 10, or 15. After the participant had walked away from the building for the designated distance, the experimenter fumbled with the filing box, spilling the box of pens onto the ground. The experimenter then proceeded to pick up the pens. The experimenter recorded whether or not the participant said thanks, whether he or she reciprocated by returning to help pick up the pens, and whether or not he or she noticed the door-holder dropping the pens, as evidenced by turning around and looking at the experimenter after he had dropped the pens. Trials were conducted at locations on campus that had glass doors and open areas of at least 25 feet of flat concrete immediately outside the door. Experimenters changed location a minimum of twice per hour. The same inter-trial interval and participant selection criteria were used as in Study I, except that no trials were run with bystanders within 50 feet of the door to minimize confounds caused by the presence of other people. Trials were aborted if another bystander entered the area, if the participant touched the door, or if ambient noise may have hindered the participant's ability to hear the pens drop. Experimenters used their own judgment to determine if the area was too noisy, based on the proximity to other people, roads, or construction equipment. Trials were not conducted near any sources of noise, and were not near other people so we are confident that the pens dropping on the concrete were heard by the participants. Trial condition was selected randomly. Statistical inference of differences in frequency of helping and verbal thanks and regression analysis of the distance participants walked back to help, was calculated using SPSS version 18.

### Results

After the experimenter held the door, 97/194 (50%) participants thanked the experimenter. The proportion of participants who thanked was greater in the high-effort condition (84.9%) than in the low-effort condition (30.5%), χ^2^(1, 194) = 58.65; *p* < 0.001.

Fifty four participants (27%) helped the experimenter pick up the pens. The proportion of participants who did so was greater for the high-effort condition (64%) than for the low-effort condition (19%), χ^2^(1, 194) = 6.569; *p* = 0.010. The proportion of participants who helped pick up the pens in the group who offered verbal thanks (29%) was not different from that in the group who did not (25%), χ^2^(1, 194) = 0.374; *p* = 0.541.

**Figure 3:**
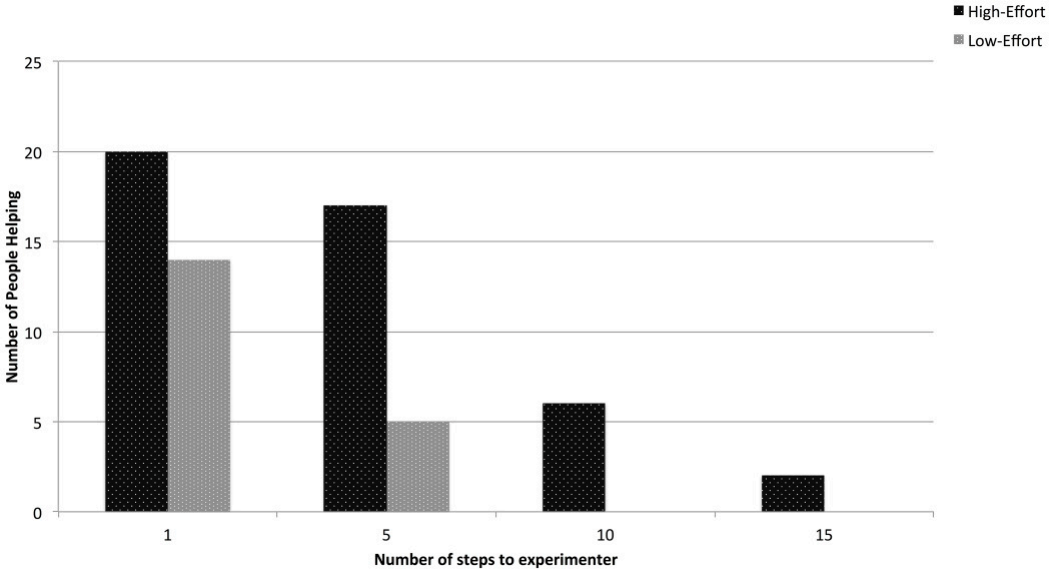
**Overall helping decreased with distance**. Individuals in the high-effort condition were statistically more likely to help than individuals in the low-effort condition at each distance.

The likelihood of participants' helping to pick up the pens varied with physical distance. The experimenter waited for the participant to travel a specified number of steps out of the building before dropping the pens. A binary logistic regression showed that the helping frequencies at 5 steps (*OR* = 0.141, *p* < 0.0001), 10 steps (*OR* = 0.049, *p* < 0.0001), and 15 steps (*OR* = 0.018, *p* < 0.0001) were smaller than the helping frequency at the 1 step distance (see Figure 3, for details). Participants in the high-effort condition helped in greater proportion at all distances when compared to the low-effort condition (See Figure 3). The meaning of this finding is limited by the small number of participants in the 10 and 15 pace distances who helped picking up pens, though only participants in the high-effort condition helped at these distances.

### Discussion

The data in Study II supported each of our predictions. Participants offered verbal thanks and reciprocated more frequently in the high-effort condition, and participants who offered verbal thanks did not help more often. Participants at every distance were more likely to help the door-holder if they were part of the high-effort condition than of the low-effort condition. Study II's results confirm previous results showing that the amount of effort put into a favor will influence reciprocal helping (Greenberg and Frisch, 1972; Goei and Boster, 2005; Tsang, 2007). Finally, the results of Study II confirm Study I's findings by demonstrating that verbal thanks do not predict helping behavior.

## General discussion

This investigation examined how effort spent holding a door leads to (a) verbal thanks, (b) changes the likelihood of reciprocal help, and (c) whether verbal thanks predict subsequent help. In both studies, the effort spent to hold the door was manipulated to change the reaction to the door-holder. In Study II, the perceived effort (holding the door while also holding a filing box) was sufficient to increase reciprocal helping toward the door-holder. In Study I, perceived effort (holding only the door) was not sufficient to create helping behavior directed toward another person. Directly comparing Study I and Study II, however, is not straightforward because they differed along a number of dimensions (i.e., stranger vs. benefactor); and it is thus difficult to know how door-holding could lead to upstream reciprocity (Nowak and Roch, 2007). The current results do, however, extend the findings of previous laboratory-based studies of reciprocity and helping behavior (Regan, 1971; Greenberg and Frisch, 1972; Tsang, 2007) by demonstrating that the effort spent to produce a small favor, such as door-holding, can modulate reciprocal helping in the real world. This also extends the notion that the gratitude we feel for a benefit we have received is tied to the relative and subjective value of the benefit itself (Wood et al., 2011).

The experiments presented here are limited by a number of variables that make interpretations of their results a challenge. It is possible, for instance, that there are differences in how we treat someone entering a building as we exit compared to someone leaving a building with us and that we are broadly more likely to help those who are entering as we leave. This confound would need to be tested directly, but suffice it to say that to whatever extent the results are hampered by this, the same concern would be present in previous reports of door-holding in which the direction of ingress or egress was not directly compared (Okamoto and Robinson, 1997; Goldman et al., 1981; McCarty and Kelly, 2013). Further, it is possible that in the low-effort conditions, the participants felt that the door-holder was being rude by cutting in front of the door and these participants' actions were influenced by the perceived slight. Their behavior may also have been motivated by empathy, as prior laboratory research has shown that perspective-taking and empathic concern can generate helping behavior (Myers et al., 2014). Likewise, while participants in the low-effort condition may have felt that the door-holder was rude, it is also possible that participants in the high-effort condition felt indebted to the door-holder, or even pitied the person, and thus only begrudgingly returned the favor. The difficulty in assessing participants' feelings after an interaction is another limitation of observational studies.

Finally, an important limitation stems from the fact that the high-effort door-holding used increased pro-social effort via eye-contact and smiling to boost the perceived genuine helpfulness of the favor, and it is unclear which aspect of the door-holder's behavior was influencing the participants' behavior. Genuine helpfulness is an important aspect of eliciting gratitude (Wood et al., 2008) and subsequent helping behavior (Tsang, 2006b). The present study was aimed more generally at identifying how the effort spent to produce a small favor could elicit forms of reciprocal helping. Future investigations should focus on how each of the factors of ingress/egress, eye-contact, type of favor, etc. each contribute independently to reciprocal helping.

In studies I and II, the effort spent to hold the door was sufficient to increase the frequency of verbal thanks toward the door holder, in support of previous findings (Okamoto and Robinson, 1997). We extend these findings by demonstrating that participants who offered verbal thanks to the door-holder were not more likely to perform reciprocal helping. Previous research has highlighted the unique role of gratitude in motivating reciprocal help apart from general positive mood (Bartlett and DeSteno, 2006). In the present study, however, the participants' feelings and motivations are unknown and we cannot claim that they felt gratitude–or any other emotion. Thus, the effects of verbal thanks may be due to participants following social norms for politeness and that verbal thanking completes the social contract of recognizing the door-holding; this is consistent with the lack of a connection between verbal thanking and reciprocal helping. However, the finding that the effort spent to hold a door predicted reciprocal helping in Study II indicates that door-holding itself can inspire notable acts of repayment.

In spite of their intrinsic challenges, studies of field behavior offer unique insight into human relationships, cognition and emotion (Patterson, 2008). In the current study, spontaneous social behavior was measured in a novel way that allows conclusions about how we perceive the things others do for us–and how we act in kind. We see for the first time that verbal thanking and reciprocal helping are not inherently correlated. Future research should investigate whether this is true across a variety of circumstances. More broadly, however, we are heartened by the many participants that, after receiving a trivial favor, spent considerable time to help a stranger.

### Conflict of interest statement

The authors declare that the research was conducted in the absence of any commercial or financial relationships that could be construed as a potential conflict of interest.
